# Quantitative EEG as a Biomarker in Evaluating Post-Stroke Depression

**DOI:** 10.3390/diagnostics13010049

**Published:** 2022-12-23

**Authors:** Livia Livinț Popa, Diana Chira, Victor Dăbală, Elian Hapca, Bogdan Ovidiu Popescu, Constantin Dina, Răzvan Cherecheș, Ștefan Strilciuc, Dafin F. Mureșanu

**Affiliations:** 1 RoNeuro Institute for Neurological Research and Diagnostic, 400364 Cluj-Napoca, Romania; 2 Department of Neuroscience, Iuliu Hatieganu University of Medicine and Pharmacy, 400083 Cluj-Napoca, Romania; 3 Department of Neuroscience, Carol Davila University of Medicine and Pharmacy, 050474 Bucharest, Romania; 4 Faculty of Medicine, Ovidius University, 900527 Constanta, Romania; 5 Department of Public Health, Babes-Bolyai University, 400294 Cluj-Napoca, Romania

**Keywords:** qEEG, ischemic stroke, post-stroke depression, HADS, DTABR, neuropsychological assessment

## Abstract

*Introduction*: Post-stroke depression (PSD) has complex pathophysiology determined by various biological and psychological factors. Although it is a long-term complication of stroke, PSD is often underdiagnosed. Given the diagnostic role of quantitative electroencephalography (qEEG) in depression, it was investigated whether a possible marker of PSD could be identified by observing the evolution of the (Delta + Theta)/(Alpha + Beta) Ratio (DTABR), respectively the Delta/Alpha Ratio (DAR) values in post-stroke depressed patients (evaluated through the HADS-D subscale). *Methods*: The current paper analyzed the data of 57 patients initially selected from a randomized control trial (RCT) that assessed the role of N-Pep 12 in stroke rehabilitation. EEG recordings from the original trial database were analyzed using signal processing techniques, respecting the conditions (eyes open, eyes closed), and several cognitive tasks. *Results*: We observed two significant associations between the DTABR values and the HADS-D scores of post-stroke depressed patients for each of the two visits (V1 and V2) of the N-Pep 12 trial. We recorded the relationships in the Global (V1 = 30 to 120 days after stroke) and Frontal Extended (V2 = 90 days after stroke) regions during cognitive tasks that trained attention and working memory. For the second visit, the association between the analyzed variables was negative. *Conclusions*: As both our relationships were described during the cognitive condition, we can state that the neural networks involved in processing attention and working memory might go through a reorganization process one to four months after the stroke onset. After a period longer than six months, the process could localize itself at the level of frontal regions, highlighting a possible divergence between the local frontal dynamics and the subjective well-being of stroke survivors. QEEG parameters linked to stroke progression evolution (like DAR or DTABR) can facilitate the identification of the most common neuropsychiatric complication in stroke survivors.

## 1. Introduction

Stroke is the second most common cause of death in individuals over 60 and the fifth major cause of mortality in people aged 15 to 59 [1]. Regarding stroke impairment, neuropsychiatric conditions are the most prevalent category among survivors. They are linked to poorer functional outcomes, decreased treatment adherence, and an increased caregiver burden [2]. Being associated with delayed recovery and increased mortality, complications like post-stroke depression (PSD) negatively impact the life quality of stroke survivors [3,4,5].

Despite being a common and long-term complication of ischemic stroke, PSD often remains underdiagnosed. Its prevalence can vary considerably, with several reports illustrating the range between 11–61% and a peak between three and six months after the ischemic event [6,7,8,9]. The link between stroke and depression has been researched for many years, demonstrating that depressive symptoms were significantly more common in patients with motor dysfunctions after ischemic events than in patients with similar physical impairments [7,10].

The Hospital Anxiety and Depression Scale (HADS) is a clinical tool consisting of 14 questions equally divided into two subscales (HADS-A for grading anxiety and HADS-D for grading depression). The HADS-S subscale has been previously used to assess PSD in several studies [4,5,11].

Processing cerebral signals by mathematical and statistical algorithms are the appanage of quantitative EEG (qEEG). Performing computations of the recorded signals can bring valuable information regarding frequency (shifting from the time domain through the Fourier Transform–FT), power spectrum density, or the connections between various brain regions (connectivity) [12].

Due to its highly accurate temporal resolution, qEEG can observe changes in the cerebral activity of patients with depression. The decline in the prefrontal region and the absence of symmetrical discharges of the combined power of alpha and theta waves were the most reliable qEEG markers observed [13,14,15]. Although specific studies have highlighted the role of qEEG in the follow-up of neuropsychiatric complications, it has not been previously used to assess PSD [16,17].

Specific qEEG ratios, like the (delta + theta)/(alpha + beta) ratio (DTABR) or the delta/alpha ratio (DAR), can help observe the evolution of ischemic stroke patients, especially in the subacute period [18,19,20]. Moreover, both DAR and DTABR can deliver meaningful information regarding the outcome of patients after an ischemic stroke [21,22,23].

The primary objective of the current paper was to describe a possible marker of PSD by analyzing the DAR and DRABR values in PSD patients (evaluated through the HADS-D subscale), additionally observing the accuracy of the subscale in the diagnosis of PSD. The data was extracted from a randomized control trial, which assessed the role of the dietary supplement N-Pep-12 in the neuro recovery of stroke by multiple outcome scales [24], as well as from a secondary data analysis of the mentioned trial [25].

## 2. Materials and Methods

### 2.1. Study Design, Population, and Procedures

The N-Pep 12 trial by Balea et al. is a prospective, double-blind, randomized controlled trial that focused on the role of N-Pep-12 (Cebrium^®^) in the neurorehabilitation of stroke survivors with cognitive disorders. It involved a multivariate approach, selecting five neuropsychological instruments for evaluating the subjects: The Montreal Cognitive Assessment, Color Trails Test—A & B, Processing Speed Index—Digit Symbol Coding and Symbol Search, Digit Span Forward and Backwards, as well as the HAD Scale [24].

The population described in the present study included 57 subjects from the RCT by Balea et al. (39 of the intervention group and 18 controls), assessed through qEEG algorithms in a 2021 secondary data analysis. In addition, demographics, medical history, EEG recordings, depression scores graded by the HADS-D subscale, and the qEEG values were collected for the current study from the same RCT and secondary analysis to identify a possible PSD biomarker [24,25].

All patients were selected based on the inclusion criteria of the original RCT [24]: the window of admission (between one to four months after the ischemic event), type of stroke (ischemic lesion), localization (supratentorial CT or MRI-established lesion), and no previous clinical and radiological signs of brain ischemia. The absence of disability before the onset (certified by a score of 0 or 1 on the Modified Rankin Scale) was considered a criterion of eligibility. The National Institutes of Health Stroke Scale (NIHSS) was selected for evaluating stroke severity.

### 2.2. EEG Signal Acquisition, Preprocessing, and Analysis 

Brain signals were recorded during both resting and cognitive conditions during the N-Pep-12 trial. The International 10-10 system for electrode placement was selected, using the Nicolet^®^ 32-channel Amplifier to register the EEG activity [24].

The cognitive conditions consisted of three neuropsychological tasks: RTI—Reaction Time Index (COG1), OTS—One Touch Stockings (COG2), and PAL—Period Associated Learning (COG3). The patient had access to a tablet to solve the cognitive tasks. The first task (RTI) assessed patients’ reaction time, processing, and psychomotor speed. The capacity of working memory, planning, and other executive functions (e.g., attention) was the subject of analysis for the second cognitive exercise (OTS). The final task (PAL) was used to test the capacity of subjects to learn novel notions. All three tasks are part of CANTAB (Cambridge Neuropsychological Test Automated Battery—Cambridge Cognition^®^) and were used to observe changes in the EEG traces while the patients train specific functions of the frontal and temporal lobes.

**Figure 1:**
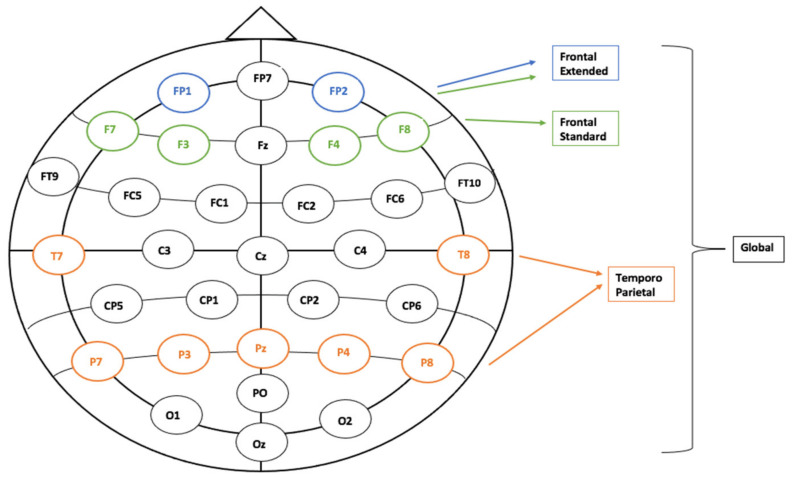
The four Regions of Interest (ROIs)—General—All 32 electrodes; Frontal Standard—4 electrodes; Frontal Extended—6 electrodes; Temporo-Parietal—7 electrodes.

Four different regions of interest (ROIs) were created per the brain areas assessed by cognitive tasks (Global—comprising all electrodes, Frontal Standard—4 electrodes, Frontal Extended—expanding the previous group to the two frontopolar electrodes, Temporo-Parietal—7 electrodes, of which P7 and P8 are considered an equivalent of T5, respectively T6) (Figure 1).

The following EEG sequence was recorded and selected for our analysis (1) Eyes open (EO1) for 5 min; (2) Eyes closed (EC1) for 5 min, (3) Cognitive tasks (COG1—RTI; COG2—OTS; COG3—PAL) for 10 min altogether (4) Eyes open (EO2) for 5 min and (5) Eyes closed (EC2) for 5 min.

The EEG data were preprocessed and analyzed using the BrainVision Analyzer (BVA) version 2.1 software (Brain Products GmbH, Gilching, Germany). Establishing the position for the 32 channels was the first step of data preprocessing, each channel being virtually placed on a 2D circular map based on the values of three parameters (radius, theta, and pi). The sampling rate (the number of times the data is collected from all the available electrodes per second) was downsized to 512 Hz from 1024 Hz. Semi-automatic Raw Data Inspection (RDI) was used to eliminate large-scale artifacts, marking an area for rejection around the artifact that starts 0.2 s before the unwanted portion and ends 0.2 s after [25].

Low-pass and high-pass filter values (0.5, respectively, 40 Hz) were selected. The notch filter was also set at 50 Hz. The continuous EEG recordings were segmented using start and end markers, recreating the original seven segments—EO1 & EC1, COG1, COG2, and COG3, and EO2 & EC2, respectively.

Semi-automatic Independent Component Analysis (ICA) was computed on all seven conditions. ICA components with kurtosis and energy values higher than 3, respectively 2, were not considered viable, as the majority of artifact segments (e.g., electrode, pop-up, salt-bridge, or gross muscular artifacts) are located above these values. A second segmentation process was applied, sorting the data into epochs of 2 s. Frequency-domain analysis was performed using the Fast-Fourier transform (FFT).

An average of the absolute power (AP for all three conditions (resting state—eyes open, resting state—eyes closed, and cognitive tasks) was computed for all 32 electrodes. The absolute power was calculated for the following power bands: δ (0.5–4 Hz), θ (4–8 Hz), α (8–13 Hz) and, β (13–30 Hz). Therefore, two ratios were determined:

DTABR (Delta Theta/Alpha Beta Ratio) = (δ + θ)/(α + β)

DAR (Delta Alpha Ratio) = δ/α

All statistical tests were two-sided. The descriptive statistics were performed using SPSS^®^ Statistics v25 (IBM^®^, Armonk, New York, USA). Pearson’s correlation was performed to determine the association between the HADS-D scores and the AP values in the frequency bands and ratios mentioned above. 

## 3. Results

Out of 57 patients, the proportion of male subjects was 82.5%. As shown in Table 1, the most prevalent type of ischemic stroke (TOAST classification) was thrombotic (80.7%), followed by cardioembolic (15.8%) and lacunar (3.5%). Regarding stroke severity, 54.4% of the cases were moderate ischemic strokes (NIHSS between 5 and 12 points). Table 1 also presents data on the vascular territory in which the stroke occurred [25].

Table 2 presents the frequency of depressive symptoms graded through the HADS-D subscale. The majority of patients had no depressive symptoms during either visit (66.7% and 75.4%), with only one patient diagnosed with severe depression (V1) [25].

One direct association stood out during the reinterpretation of the V1 results (Baseline—90 to 120 days after stroke onset), linking the level of depression scored on the HADS-D test and the DTAB Ratio value (Table 3). In addition, the association is observed globally (across all 32 electrodes) during the second and third cognitive tasks.

A second significant association involving the DTABR values and the HADS-D scores was identified for the second visit during the second and third cognitive tasks (Table 4). This finding was identified in the Frontal Extended region as a negative correlation.

No statistically significant association was found at any of the time points (V1 and V2) for the first cognitive task (COG1) in either of the two regions mentioned above (Table 5).

The analysis for both time points (V1 and V2) produced no any additional significant correlations for neither ratio (DTABR or DAR).

As significant results were found only in the Global and Frontal Extended regions, Table 6 presents the DTABR scores calculated from the averaged AP values. The AP was computed for the delta, theta, alpha and beta frequency bands at each visit (V1 and V2) for all cognitive task conditions (COG1, COG2, and COG3).

## 4. Discussion

The current study aimed to explore a possible PSD marker by looking at the behavior of two qEEG parameters (DTABR and DAR) in a population of stroke survivors graded for depression using the HADS-D subscale [24,25].

Almost four decades ago, it was first described that delta activity registers a peak throughout the infarcted area in relationship with the reduction of blood supply in that region. Previous studies discovered that before an increase in delta activity can be recognized, the general attenuation of both alpha and beta waves might be observed. Therefore, the DTABR can be summarized as a predominance of slow frequencies (delta or/and theta) over fast ones (alpha or/and beta). If the values of the ratio increase over time, the overall prognostic can be considered unfavorable [26,27,28].

A study by Finnigan et al. [17] observed changes in the delta frequency band (AP values) for acute stroke patients that varied with time. A significant association between the NIHS Scale and delta activity 30 days after stroke onset was validated for is chemic stroke survivors [17]. Moreover, a paper by Burghaus et al. [29] demonstrated the widespread presence of delta activity paired with a decrease in the alpha frequency band for patients with malignant evolution after brain ischemia (e.g., massive cerebral edema). Regarding mild and moderate strokes (NIHSS between 0–12), the absence of delta waves and predominance of fast frequencies (e.g., beta) translated to a more favorable outcome [29].

The literature regarding PSD evaluation through EEG techniques is relatively scarce, with contrasting results between studies. As PSD subjects exhibit increased power in the delta and theta bands, elevated alpha and beta activity were found in patients without PSD, translated as a decreased general slowing and decreased DTABR values [30]. One study highlighted the predominance of alpha and theta activity specifically in PSD patients [31], alongside other papers that observed that greater levels of alpha power in the left frontal region could be associated with depressive symptoms and an elevated tendency toward general depression [30,32].

While the positive correlation was identified during V1 (one to four months after the stroke), finding a negatively correlated relation during follow-up (90 days after the first assessment) in one single ROI (Frontal Extended) could orientate us towards the reorganization of the communication between the lesionated area and the other cortical regions [33].

A study by Veer et al. [34], which explored the functional connectivity (FC) of the whole brain in patients with major depression during resting state, found changes in the FC in the left frontal region, part of a neural network implied in attention and working memory. Moreover, areas of the same network were found to be activated during any action that would require cognitive effort [34].

The second cognitive task (OTS—One Touch Stockings) evaluated attention and working memory as executive processes. As both identified relationships were significant during the cognitive condition, in which the network system described by Veer et al. would express high levels of activity, an inceptive reorganizational phase of the neuronal activity could be observed at V1. In the end, the process could localize itself in the frontal part of the brain at V2 (the period between the two visits being three months) [35].

A 2015 paper by Zheng et al. [31] assessed neural complexity (the level of complexity of a specific neural system) between ischemic stroke survivors with PSD, without PSD, and controls. The study used the Lempel-Ziv Complexity (LZC) to evaluate changes in the recordings of the three groups. A decreased neural complexity was observed in PSD patients, especially in the frontal and temporal regions of stroke survivors with increased severity of PSD. In addition, the presence of slow waves (especially theta) was reported [31].

A divergence between the local frontal dynamics and the actual status of stroke survivors could arise. Although the HADS-D scale is a simple and facile way of grading depression, our findings could suggest a decreased accuracy for identifying symptoms in PSD patients. While the patient could be categorized as without depressive symptoms, a localized slowing in the frontal brain area should be observed through an elevated DTAB ratio. The possible misdiagnosing through the appliance of the HADS-D subscale could contribute to the uncertainty wandering around ischemic-related neuropsychiatric conditions.

Considering the different conditions during which data was acquired (eyes open, eyes closed, and cognitive tasks), our study’s methodology is distinguished within the available literature. However, several limitations can be pointed out, including the descriptive nature of the study, the number of analyzed subjects, and the reduced number of patients diagnosed with depression.

## 5. Conclusions

To our knowledge, no previous studies investigating the value of qEEG ratios (DAR and DTABR) in PSD were identified. The present article could aid the establishment of qEEG as a technique for diagnosing PSD in the future. Regardless of its pure descriptive nature, the current study could provide valuable information on the relationship between the frequency bands in the power spectrum. Furthermore, DTABR could be used to assess the predominance of either slow or fast waves in ischemic stroke patients.

While the HADS-D subscale is a general approach for measuring depression, it is unwise to consider it a standard in diagnosing PSD. As our results show, most of the patients evaluated through HADS-D were described as depression free. Additional research on the role of DTABR and other qEEG parameters in PSD is recommended, using RCT designs to encompass larger populations.

The absence of a standardized assessment to assess PSD and the scarce and contrasting literature on the subject are the reasons why our attention should focus on identifying a marker for the condition. While qEEG is not as prevalent as other investigations from the field of neurology, the method could help diagnose post-stroke neuropsychiatric comorbidities in the future.

## Figures and Tables

**Table 1:** Patients’ characteristics and demographics.

Demographic Categories	Frequency	Valid Percentage
**Gender**		
Female	10	17.5%
Male	47	82.5%
**Age group**		
37–60	24	42.1%
61–79	33	57.9%
**Type of stroke**		
Thrombotic	46	80.7%
Lacunar	2	3.5%
Cardioembolic	9	15.8%
**Stroke severity**		
Minor (NIHSS 1–4 points)	22	38.6%
Moderate (NIHSS 5–12 points)	31	54.4%
Mild to Severe (NIHSS 17–20 points)	4	7.0%
**Vascular Territory**		
Left Middle Cerebral Artery	28	49.1%
Right Middle Cerebral Artery	25	43.9%
Left Posterior Cerebral Artery	3	5.3%
Right Posterior Cerebral Artery	1	1.8%

**Table 2:** HADS-D Scores—depressive symptoms.

	VISIT 1		VISIT 2	
	Frequency	Percentage	Frequency	Percentage
No depressive symptoms (<7)	38	66.7%	43	75.4%
Mild (8–10)	13	22.8%	11	19.3%
Moderate (11–14)	5	8.8%	3	5.3%
Severe (15–21)	1	1.8%	0	0.0%

**Table 3:** Significant correlations at V1.

Analysis	Time Point	Region	Sub-Type	Correlation Coefficient	Significance
DTABR COG HADS-D	V1	Global	COG2	0.292	0.028
			COG3	0.202	0.027

**Table 4:** Significant correlations at V2.

Analysis	Time Point	Region	Sub-Type	Correlation Coefficient	Significance
DTABR COG HADS-D	V2	Frontal Extended	COG2	−264	0.049
			COG3	−392	0.003

**Table 5:** Non-significant correlations.

Analysis	Time Point	Region	Sub-Type	Correlation Coefficient	Significance
DTABR COG HADS-D	V1	Global	COG1	−002	0.988
	V2	Frontal Extended		−119	0.383

**Table 6:** DTAB Ratio Scores.

DTAB Ratio Scores	Visit 1			Visit 2		
	COG1	COG2	COG3	COG1	COG2	COG3
Global	1.516	1.500	1.440	1.508	1.468	1.432
Frontal Extended	1.495	1.478	1.425	1.486	1.469	1.418

## Data Availability

Not applicable.
